# *Paracoccidioides* Species Complex: Ecology, Phylogeny, Sexual Reproduction, and Virulence

**DOI:** 10.1371/journal.ppat.1004397

**Published:** 2014-10-30

**Authors:** Marcus M. Teixeira, Raquel C. Theodoro, Gustavo Nino-Vega, Eduardo Bagagli, Maria S. S. Felipe

**Affiliations:** 1 Departamento de Biologia Celular, Universidade de Brasília (UnB), Brasília, Brazil; 2 Departamento de Biologia Celular e Genética, Universidade Federal do Rio Grande do Norte (UFRN), Natal, Brazil; 3 Centro de Microbiología y Biología Celular, Instituto Venezolano de Investigaciones Científicas (IVIC), Caracas, Venezuela; 4 Departamento de Microbiologia e Imunologia, Universidade Estadual Paulista Júlio de Mesquita Filho (UNESP), Botucatu, Brazil; 5 Pós-Graduação em Ciências Genômicas e Biotecnologia, Universidade Católica de Brasília, Brasília, Brazil; Duke University Medical Center, United States of America

## The *Paracoccidioides* Genus and Paracoccidioidomycosis

Paracoccidioidomycosis (PCM) is a deep systemic mycosis caused by human fungal pathogens of the *Paracoccidioides* genus. The disease is geographically restricted to subtropical areas of Latin America (from south of Mexico to north of Argentina) with a high prevalence in Brazil, Colombia, Venezuela, and Argentina [1]. The annual incidence rate in Brazil is 10–30 infections per million inhabitants, and the mean mortality rate is 1.4 per million inhabitants per year, making this disease the highest cause of mortality among systemic mycoses [2]. PCM is endemic in rural populations and mainly affects individuals engaged in agricultural activities, who inhale aerosols containing fungal material during manipulation of the soil.

**Figure 1:**
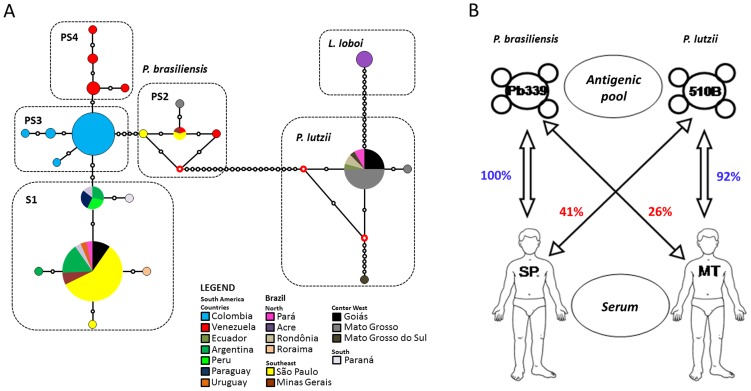
(A) Median-joining haplotypic network distribution of the *Paracoccidioides* genus and *L. loboi* based on the *gp43* marker. The size of the circumference is proportional to the haplotype frequency, and colors vary according to the sampling location of each haplotype. Red dots (median vectors) are hypothetical missing intermediates, and black dots represent each mutation site. (B) Schematic representation of serum/antigen compatibility among patients and isolates from Mato Grosso and São Paulo used in serological tests. The illustration shows the low immunogenic specificity when serum and antigenic pool from different states are reacted [41].

Molecular evolutionary studies place the genus *Paracoccidioides* in the thermodimorphic fungal pathogen clade related to the family Ajellomycetaceae (Ascomycetes), which includes the *Blastomyces*, *Histoplasma*, and *Emmonsia* genera, and with which it shares a common ancestor, *Lacazia loboi*. PCM can be caused by two species *Paracoccidioides brasiliensis* and *P. lutzii* [3]. *P. brasiliensis* has been considered a single species since its discovery, although several studies including molecular and morphological data support the split of *P. brasiliensis* into two species [3], [4]. *P. lutzii* is composed of a single monophyletic and recombining population so far found in central, southwest, and north Brazil and Ecuador [3]–[5]. On the other hand, *P. brasiliensis* contains a complex of at least four different cryptic species (S1, PS2, PS3 and PS4; Figure 1A [6]). *P. brasiliensis* S1 represents a monophyletic and recombining population widely distributed in South America and has been associated with the majority of cases of PCM detected up until the present time. Strains belonging to *P. brasiliensis* S1 have previously been recovered from armadillos, soil, and penguin feces [6]. *P. brasiliensis* PS2 is a paraphyletic and recombining population identified so far only in Brazil and Venezuela [6]. *P. brasiliensis* PS3 is comprised of a monophyletic and clonal population that has been recovered in humans and armadillos in endemic regions of Colombia [6]. *P. brasiliensis* PS4 was recently identified and is composed of a monophyletic population of clinical isolates from Venezuela [5], [7]. Besides the typical bicorn cocked hat– and barrel-shaped conidia produced by both species, *P. lutzii* frequently produces elongated rod-shaped conidia, a characteristic feature that may be used for species identification [3]. Because of the difficulties of conidia production in the laboratory and slight morphological differences among species, molecular identification of *Paracoccidioides* species has become the most common tool of choice. Several molecular markers have already been applied in population studies of the *Pararacoccidioides* genus, and for multilocus sequencing typing, *gp43*, *arf*, *β-tub*, and *hsp70* loci are the best choices for species delineation [4], [6].

## Phylogeography of the *Paracoccidioides* Genus

The finding of the cryptic species in the genus *Paracoccidioides* has led us to explore the evolutionary mechanisms that were responsible for the current geographic distribution of its five phylogenetic species (S1, PS2, PS3, PS4, and *P. lutzii*). Phylogeographic inferences from three different loci revealed simultaneous geographic expansions of S1 isolates. This represents a dispersal by distance, leading to a nonsexual population (PS3) that does not produce any gene flow between other species, and a long-distance colonization or a fragmentation resulting in the separation of Venezuelan PS4 species [3]–[5], [7]. The dispersal event that resulted in PS3 has been confirmed, with the remaining divergence processes due to vicariance events. Despite the great stability of the Guiana and Brazilian shields, the uplift of the Andes and episodic marine inclusions 61 million years ago, 20 million years ago, and 11.8–7.0 million years ago may have favored vicariance between *Paracoccidioides* and *Lacazia*. This may have occurred by creating new and empty ecological niches represented by wetlands and/or totally submerged areas, as well as by the simultaneous emergence of riverine Cetacean mammals. The recent dispersal of PS3 to Colombia may also be explained by the complete submersion of Colombian territory by the Pebas/Solimões lake, derived from marine incursions in the late Miocene era [8] and indicating a recent occupation. As there are no clear geographic barriers, the most challenging task is to explain the speciation processes that have given rise to S1, PS2, and *P. lutzii* in the very stable Brazilian shield. Indeed, the prevalence of *P. lutzii* in central-western Brazil and its relatively close proximity to the S1 and PS2 occurrence areas suggests a parapatric speciation. With regards to the divergence between S1 and PS2 and the current sympatry observed between them, probable differences in preferences for substrates and resources in their saprobe lifestyle that might have triggered a disruptive selection should be considered [5]. However, more studies are required to complete the biogeographical puzzle of the *Paracoccidioides* genus. These include mapping of the cryptic species in environmental, rather than clinical, samples, as well as searching for saprobe differences between sympatric species.

## Ecology of *P. brasiliensis* and *P. lutzii*

Onygenalean (Ascomycota) organisms have typically evolved by adapting to two distinct ecological niches, the first represented by saprobic conditions in soil and the second by the live tissues of animal hosts. Genomic adaptations such as loss of carbohydrate-degrading enzymes, gain of proteases, and the ability to produce infective conidia allowing long association with the mammalian host are more adapted to a biotrophic lifestyle [9], [10]. Epidemiological evidence indicates that the saprobic forms of *P. brasiliensis* and *P. lutzii* may occur in some restricted and/or protected soil conditions, in places containing natural and anthropic disturbed vegetation near water sources [11]. Isolation of *P. brasiliensis* directly from its saprobic form has proved to be difficult. However, the fungus has been repeatedly cultured from the armadillo species *Dasypus novemcinctus* and *Cabassous centralis* in endemic PCM areas [12], [13] and, in unique cases, from dogs and two-toed sloths [14], [15]. Additional evidence of the infection of several wild and domestic animals has also been provided by intradermal, serological, histopathological, and molecular tests, revealing a broad distribution and adaptation to mammalian hosts [16].

Although outbreaks of PCM have not been documented, the geographical distribution of the disease is heterogeneous and is associated with moderate-to-high precipitation rates, mild temperatures, and fertile soils. The disease occurs in areas such as the central-western and northern regions of Brazil where agricultural activities are more commonly employed. Climatic anomalies, such as those triggered by the 1982–1983 El Niño event, have been associated with an excess of acute PCM cases when compared with the number of expected cases for the same period. This indicates the presence of a temporal cluster of the disease in the state of São Paulo, Brazil, occurring in the year 1985 [17]. Climatic conditions resulting in an atypical increase in soil water storage in 1982–1983 and in an increase in the absolute air humidity in 1984 may have contributed firstly to fungus growth and then to conidial dispersal. This evidently follows the “grow and blow” model already proposed for coccidioidomycosis outbreaks [18]. Experimental studies have indicated that the several genetic groups or cryptic species of *P. brasiliensis* have different abilities in producing the infective conidia, and this may in turn produce differential rates of infection. For example, the S1 and PS2 sympatric cryptic species of *P. brasiliensis* occur at a disproportional rate of approximately 9∶1 in both patient and armadillo isolates. At the same time, isolates of the S1 genotype produce many more conidia than PS2 isolates [5].

## Reproductive Modes in the *Paracoccidioides* Species Complex

*Paracoccidioides* was considered an asexual and clonal microorganism for many years [1]. The anamorph of *Paracoccidioides* is characterized by multiple budding yeast cells that grow at 37°C in mammalian tissues or by mycelia that produce chlamydospores or conidia at 25°C in the environment. Recently, population genetics and comparative genomic studies have provided evidence for different breeding strategies in the *Paracoccidioides* genus. Recombination events were detected in both *P. lutzii* and *P. brasiliensis* (S1 and PS2), and the *P. brasiliensis* PS3 population was considered clonal [4], [6]. The mating type locus was identified in the three sequenced genomes, and a single copy of *MAT1-1* or *MAT1-2* was found, thus suggesting a bipolar mating system [9]. According to Torres et al. [19], *MAT* gene distribution was evaluated with regards to the country of origin and phylogenetic species, revealing a 1∶1 ratio of *MAT1-1* and *MAT1-2*. Additionally, Teixeira et al. [20] tested the *MAT* gene distribution in 98 *Paracoccidioides* isolates and revealed a slight (2-fold) prevalence of the *MAT1-2* idiomorph. Unexpectedly, both the *MAT1-1* and *MAT1-2* genes were identified in 13 of the clinical isolates, suggesting that homothallism may exist in the *Paracoccidioides* genus.

Orthologs of mating and meiotic regulators that have been well characterized in a wide range of fungi were found by comparative genomics to be highly conserved in *Paracoccidioides* and other Ajellomycetacean sexual fungi [9], [20]. The biological functionality of α-pheromone and its receptor was elucidated using heterologous expression of these *Paracoccidioides* genes in the corresponding *Saccharomyces cerevisiae* null mutants [21]. Features related to sexual reproduction, such as coiled constricted hyphae and knob-like structures, were observed in *Paracoccidioides* species, indicating the formation of young ascocarps. In addition, multiple nuclei were found in coiled constricted hyphae, possibly as a consequence of nuclear migration during mating. Unfortunately, no cleistothecium or ascus production has so far been detected. The presence and expression of sexual machinery as well the ability to produce sex-related structures indicates that mating may occur in the *Paracoccidioides* life cycle.

## Virulence Factors Associated with Dimorphism and Host Adaptation

The most probable environmental habitat of mycelial-phase *Paracoccidioides* is the soil. Once conidia or mycelia fragments are inhaled into the lung alveoli, the fungus shifts its morphology to a yeast phase because of temperature, hormones, and immune response. This step is crucial for the survival and maintenance of *Paracoccidioides* and other dimorphic fungi in hosts. The dimorphic transition promotes changes in the cell wall composition and carbohydrate polymer structure. Additionally, the presence of an outermost layer of α-1,3-glucan in the *P. brasiliensis* yeast cell wall has been proposed as a protective shield against host defense [22], [23].

The yeast transcriptional profile indicating the diversion of pyruvate from the glycolytic pathway into the glyoxylate cycle is consistent with a lower oxygen level in infected tissues [24]. Stress adaptations that induce genes encoding molecular chaperones, such as heat shock proteins (HSPs), are a common trait of *Paracoccidioides* during dimorphism and exposition to different host niches. These adaptations may be indispensable for fungal virulence upon infection [25]. In contrast, some HSP genes are down-regulated when mycelia cells are incubated in vitro with estradiol or human female serum, in some part explaining the predominance of the disease in adult males [26]. The ability of fungal cells to adhere to host cells is also vital for the initial steps of the infection process. Phospholipase B, involved in the fungus-macrophage interaction, and PbHad32p, a hydrolase involved in adherence to host cells, have both been proposed to be important for the initial steps of the virulence process [27], [28]. The genes encoding glyceraldehyde-3-phosphate-dehydrogenase (GAPDH), identified as a cell wall–associated host-adhesion molecule in *Paracoccidioides*, and enolase, which binds to fibronectin, have also been linked to the initial adhesion of the fungus to lung epithelial cells and alveolar macrophages upon infection [29]. New strategies for gene function studies using antisense RNA technology have recently emerged, overcoming problems with gene knockout due to multinucleated cells and unstable transformants [30]. The depletion of *P. brasiliensis*–encoding genes Cdc42 [31], AOX [32], Gp43 [33], SCONC [34], HSP90 [35], P27 [36], and Rbt5 [37] contributed significantly to the elucidation of host-pathogen interaction, pathogen resistance, and virulence in those species.

In years to come, more information on virulence processes will emerge from the accumulated data from transcriptome and complete genome releases at our disposal. This will pave the way for the identification of possible mechanisms to control the initial steps of infection which, when available to clinicians, will benefit patients.

## *Paracoccidioides* Species Complex and Its Impact on Clinical and Serological Aspects of PCM

Since the discovery of the cryptic speciation in the genus *Paracoccidioides*, some important regional features of the disease were discussed regarding its impact on the current statement of PCM. The acute or subacute ( juvenile PCM) and chronic (adult PCM) are the two main forms of the disease; however, the presentation and course of the disease may vary from case to case [16]. The first discrimination between the PCM pathology related to geographical origin was observed by Barbosa et al. [38], in which patients from the central region of Brazil had predominantly lymphoabdominal forms not shared by patients from the south and southeast, a finding later confirmed by Andrade [39]. Is there a possibility that lymphoabdominal forms are associated with the pathology caused by *P. lutzii* and not by *P. brasiliensis*? Are there different pathologies caused by different species of *Paracoccidioides*? This possibility should not be ruled out and must be fully investigated in order to verify possible associations of cryptic species and different clinical manifestations of PCM. In addition to clinical manifestation, issues addressed to treatment have been raised by Hahn et al. [40] who found that patients infected with *P. lutzii* had good responses to trimethoprim-sulfamethoxazole while those infected with *P. brasiliensis* relapsed with the same drug administration. Moreover, there are known to be a high number of patients coming from the north-central region of Brazil with PCM with low or no immunoreactivity [41], [42]. Immunodiffusion tests with antigens produced by isolates from São Paulo (*P. brasiliensis* strain 339) crossed with sera from patients of Mato Grosso have low positivity (Figure 1B). Recently, serological tests confirm that sera from patients with PCM due to *P. lutzii* are able to recognize cell-free antigens from *P. lutzii*; however, sera from patients with PCM due to *P. brasiliensis* could not recognize any *P. lutzii* antigens [43]. Undoubtedly, these issues are critical and need an urgent mobilization to improve the methods of diagnosis and therapy that can specifically detect and effectively combat the *Paracoccidioides* species of a given patient with PCM.

## References

[1] San-Blas G, Nino-Vega G, Iturriaga T (2002) Paracoccidioides brasiliensis and paracoccidioidomycosis: molecular approaches to morphogenesis, diagnosis, epidemiology, taxonomy and genetics. Med Mycol 40: 225–242. 12146752 10.1080/mmy.40.3.225.242

[2] Coutinho ZF, Silva D, Lazera M, Petri V, Oliveira RM, et al (2002) Paracoccidioidomycosis mortality in Brazil (1980–1995). Cad Saude Publica 18: 1441–1454. 12244377 10.1590/s0102-311x2002000500037

[3] Teixeira MD, Theodoro RC, Oliveira FF, Machado GC, Hahn RC, et al (2013) Paracoccidioides lutzii sp. nov.: biological and clinical implications. Med Mycol 52: 19–28. 10.3109/13693786.2013.794311 23768243

[4] Teixeira MM, Theodoro RC, de Carvalho MJ, Fernandes L, Paes HC, et al (2009) Phylogenetic analysis reveals a high level of speciation in the Paracoccidioides genus. Mol Phylogenet Evol 52: 273–283. 19376249 10.1016/j.ympev.2009.04.005

[5] Theodoro RC, Teixeira Mde M, Felipe MS, Paduan Kdos S, Ribolla PM, et al (2012) Genus paracoccidioides: Species recognition and biogeographic aspects. PLoS ONE 7: e37694. 22666382 10.1371/journal.pone.0037694 PMC3364295

[6] Matute DR, McEwen JG, Puccia R, Montes BA, San-Blas G, et al (2006) Cryptic speciation and recombination in the fungus Paracoccidioides brasiliensis as revealed by gene genealogies. Mol Biol Evol 23: 65–73. 16151188 10.1093/molbev/msj008

[7] Salgado-Salazar C, Jones LR, Restrepo A, McEwen JG (2010) The human fungal pathogen Paracoccidioides brasiliensis (Onygenales: Ajellomycetaceae) is a complex of two species: phylogenetic evidence from five mitochondrial markers. Cladistics 26: 12. 10.1111/j.1096-0031.2010.00307.x 34879597

[8] Wesselingh FP, Salo JA (2006) A Miocene perspective on the evolution of the Amazonian biota. Scripta Geol 133: 19.

[9] Desjardins CA, Champion MD, Holder JW, Muszewska A, Goldberg J, et al (2011) Comparative genomic analysis of human fungal pathogens causing paracoccidioidomycosis. PLoS Genet 7: e1002345. 22046142 10.1371/journal.pgen.1002345 PMC3203195

[10] Sharpton TJ, Stajich JE, Rounsley SD, Gardner MJ, Wortman JR, et al (2009) Comparative genomic analyses of the human fungal pathogens Coccidioides and their relatives. Genome Res 19: 1722–1731. 19717792 10.1101/gr.087551.108 PMC2765278

[11] Restrepo A, McEwen JG, Castaneda E (2001) The habitat of Paracoccidioides brasiliensis: how far from solving the riddle? Med Mycol 39: 233–241. 11446526 10.1080/mmy.39.3.233.241

[12] Bagagli E, Sano A, Coelho KI, Alquati S, Miyaji M, et al (1998) Isolation of Paracoccidioides brasiliensis from armadillos (Dasypus noveminctus) captured in an endemic area of paracoccidioidomycosis. Am J Trop Med Hyg 58: 505–512. 9574800 10.4269/ajtmh.1998.58.505

[13] Corredor GG, Castano JH, Peralta LA, Diez S, Arango M, et al (1999) Isolation of Paracoccidioides brasiliensis from the nine-banded armadillo Dasypus novemcinctus, in an endemic area for paracoccidioidomycosis in Colombia. Rev Iberoam Micol 16: 216–220. 18473551

[14] de Farias MR, Condas LA, Ribeiro MG, Bosco Sde M, Muro MD, et al (2011) Paracoccidioidomycosis in a dog: case report of generalized lymphadenomegaly. Mycopathologia 172: 147–152. 21424604 10.1007/s11046-011-9412-z

[15] Trejo-Chavez A, Ramirez-Romero R, Ancer-Rodriguez J, Nevarez-Garza AM, Rodriguez-Tovar LE (2011) Disseminated paracoccidioidomycosis in a Southern two-toed sloth (Choloepus didactylus). J Comp Pathol 144: 231–234. 20961559 10.1016/j.jcpa.2010.08.012

[16] Bocca AL, Amaral AC, Teixeira MM, Sato PK, Shikanai-Yasuda MA, et al (2013) Paracoccidioidomycosis: eco-epidemiology, taxonomy and clinical and therapeutic issues. Future Microbiol 8: 1177–1191. 24020744 10.2217/fmb.13.68

[17] Barrozo LV, Benard G, Silva ME, Bagagli E, Marques SA, et al (2010) First description of a cluster of acute/subacute paracoccidioidomycosis cases and its association with a climatic anomaly. PLoS Negl Trop Dis 4: e643. 20361032 10.1371/journal.pntd.0000643 PMC2846938

[18] Tamerius JD, Comrie AC (2011) Coccidioidomycosis incidence in Arizona predicted by seasonal precipitation. PLoS ONE 6: e21009. 21701590 10.1371/journal.pone.0021009 PMC3118810

[19] Torres I, Garcia AM, Hernandez O, Gonzalez A, McEwen JG, et al (2010) Presence and expression of the mating type locus in Paracoccidioides brasiliensis isolates. Fungal Genet Biol 47: 373–380. 19932183 10.1016/j.fgb.2009.11.005

[20] Teixeira Mde M, Theodoro RC, Derengowski Lda S, Nicola AM, Bagagli E, et al (2013) Molecular and morphological data support the existence of a sexual cycle in species of the genus Paracoccidioides. Eukaryot Cell 12: 380–389. 23125354 10.1128/EC.05052-11 PMC3629771

[21] Gomes-Rezende JA, Gomes-Alves AG, Menino JF, Coelho MA, Ludovico P, et al (2012) Functionality of the Paracoccidioides mating alpha-pheromone-receptor system. PLoS ONE 7: e47033. 23056569 10.1371/journal.pone.0047033 PMC3464258

[22] Rappleye CA, Goldman WE (2006) Defining virulence genes in the dimorphic fungi. Annu Rev Microbiol 60: 281–303. 16753032 10.1146/annurev.micro.59.030804.121055

[23] San-Blas G, San-Blas F, Serrano LE (1977) Host-parasite relationships in the yeastlike form of Paracoccidioides brasiliensis strain IVIC Pb9. Infect Immun 15: 343–346. 844899 10.1128/iai.15.2.343-346.1977 PMC421372

[24] Derengowski LS, Tavares AH, Silva S, Procopio LS, Felipe MS, et al (2008) Upregulation of glyoxylate cycle genes upon Paracoccidioides brasiliensis internalization by murine macrophages and in vitro nutritional stress condition. Med Mycol 46: 125–134. 18324491 10.1080/13693780701670509

[25] Felipe MS, Torres FA, Maranhao AQ, Silva-Pereira I, Pocas-Fonseca MJ, et al (2005) Functional genome of the human pathogenic fungus Paracoccidioides brasiliensis. FEMS Immunol Med Microbiol 45: 369–381. 16061364 10.1016/j.femsim.2005.05.013

[26] Shankar J, Wu TD, Clemons KV, Monteiro JP, Mirels LF, et al (2011) Influence of 17beta-estradiol on gene expression of Paracoccidioides during mycelia-to-yeast transition. PLoS ONE 6: e28402. 22194832 10.1371/journal.pone.0028402 PMC3237447

[27] Soares DA, de Andrade RV, Silva SS, Bocca AL, Soares Felipe SM, et al (2010) Extracellular Paracoccidioides brasiliensis phospholipase B involvement in alveolar macrophage interaction. BMC Microbiol 10: 241. 20843362 10.1186/1471-2180-10-241 PMC2949701

[28] Hernandez O, Almeida AJ, Tamayo D, Torres I, Garcia AM, et al (2012) The hydrolase PbHAD32 participates in the adherence of Paracoccidioides brasiliensis conidia to epithelial lung cells. Med Mycol 50: 533–537. 21988701 10.3109/13693786.2011.619583

[29] Barbosa MS, Bao SN, Andreotti PF, de Faria FP, Felipe MS, et al (2006) Glyceraldehyde-3-phosphate dehydrogenase of Paracoccidioides brasiliensis is a cell surface protein involved in fungal adhesion to extracellular matrix proteins and interaction with cells. Infect Immun 74: 382–389. 16368993 10.1128/IAI.74.1.382-389.2006 PMC1346668

[30] Menino JF, Almeida AJ, Rodrigues F (2012) Gene knockdown in Paracoccidioides brasiliensis using antisense RNA. Methods Mol Biol 845: 187–198. 22328375 10.1007/978-1-61779-539-8_12

[31] Almeida AJ, Cunha C, Carmona JA, Sampaio-Marques B, Carvalho A, et al (2009) Cdc42p controls yeast-cell shape and virulence of Paracoccidioides brasiliensis. Fungal Genet Biol 46: 919–926. 19686860 10.1016/j.fgb.2009.08.004

[32] Ruiz OH, Gonzalez A, Almeida AJ, Tamayo D, Garcia AM, et al (2011) Alternative oxidase mediates pathogen resistance in Paracoccidioides brasiliensis infection. PLoS Negl Trop Dis 5: e1353. 22039556 10.1371/journal.pntd.0001353 PMC3201906

[33] Torres I, Hernandez O, Tamayo D, Munoz JF, Leitao NP Jr, et al (2013) Inhibition of PbGP43 expression may suggest that gp43 is a virulence factor in Paracoccidioides brasiliensis. PLoS ONE 8: e68434. 23874627 10.1371/journal.pone.0068434 PMC3708949

[34] Menino JF, Saraiva M, Gomes-Rezende J, Sturme M, Pedrosa J, et al (2013) P. brasiliensis virulence is affected by SconC, the negative regulator of inorganic sulfur assimilation. PLoS ONE 8: e74725. 24066151 10.1371/journal.pone.0074725 PMC3774720

[35] Tamayo D, Munoz JF, Torres I, Almeida AJ, Restrepo A, et al (2013) Involvement of the 90 kDa heat shock protein during adaptation of Paracoccidioides brasiliensis to different environmental conditions. Fungal Genet Biol 51: 34–41. 23207691 10.1016/j.fgb.2012.11.005

[36] Torres I, Hernandez O, Tamayo D, Munoz JF, Garcia AM, et al (2014) Paracoccidioides brasiliensis PbP27 gene: knockdown procedures and functional characterization. FEMS Yeast Res 14: 270–280. 24118983 10.1111/1567-1364.12099

[37] Bailao EF, Parente JA, Pigosso LL, de Castro KP, Fonseca FL, et al (2014) Hemoglobin uptake by Paracoccidioides spp. is receptor-mediated. PLoS Negl Trop Dis 8: e2856. 24831516 10.1371/journal.pntd.0002856 PMC4022528

[38] Barbosa W, Daher R, Oliveira AR (1968) Lymphatic abdominal forms of South American blastomycosis. Rev InstMed Trop São Paulo 10: 12. 5683034

[39] Andrade ALSS (1983) Paracoccidoidomycosis. The contribution to the study of lymphatic-abdominal form. Rev Patol Trop 12: 91.

[40] Hahn RC, Macedo AM, Fontes CJ, Batista RD, Santos NL, et al (2003) Randomly amplified polymorphic DNA as a valuable tool for epidemiological studies of Paracoccidioides brasiliensis. J Clin Microbiol 41: 2849–2854. 12843011 10.1128/JCM.41.7.2849-2854.2003 PMC165335

[41] Batista J Jr, de Camargo ZP, Fernandes GF, Vicentini AP, Fontes CJ, et al (2010) Is the geographical origin of a Paracoccidioides brasiliensis isolate important for antigen production for regional diagnosis of paracoccidioidomycosis? Mycoses 53: 176–180. 19422525 10.1111/j.1439-0507.2008.01687.x

[42] Queiroz Junior LD, de Camargo ZP, Tadano T, Rodrigues AM, Takarara DT, et al (2014) Serological and antigenic profiles of clinical isolates of Paracoccidioides spp. from Central Western Brazil. Mycoses 57: 466–72. 24635832 10.1111/myc.12183

[43] Gegembauer G, Araujo LM, Pereira EF, Rodrigues AM, Paniago AM, et al (2014) Serology of Paracoccidioidomycosis Due to Paracoccidioides lutzii. PLoS Negl Trop Dis 8: e2986. 25032829 10.1371/journal.pntd.0002986 PMC4102441

